# First report of *Lihan Tick* virus (*Phlebovirus, Phenuiviridae*) in ticks, Colombia

**DOI:** 10.1186/s12985-020-01327-9

**Published:** 2020-05-05

**Authors:** Yesica López, Jorge Miranda, Salim Mattar, Marco Gonzalez, Joel Rovnak

**Affiliations:** 1 Instituto de Investigaciones Biológicas del Trópico, Facultad de Medicina Veterinaria y Zootecnia, Universidad de Córdoba, Montería, Colombia; 2 Department of Microbiology, Immunology, and Pathology, Colorado State University, Fort Collins, CO USA

**Keywords:** *Phlebovirus*, Tick-borne diseases, Epidemiology, Zoonotic

## Abstract

**Background:**

Tick-borne phenuivirus (TBPVs) comprise human and animal viruses that can cause a variety of clinical syndromes ranging from self-limiting febrile illness to fatal haemorrhagic fevers.

**Objective:**

Detect *Phlebovirus* (Family Phenuiviridae) in ticks collected from domestic animals in Córdoba, Colombia.

**Methods:**

We collected 2365 ticks from domestic animals in three municipalities of the Department of Cordoba, Colombia in 2016. Ticks were identified and pooled by species for RNA extraction. A nested real-time PCR with specific primers for *Phlebovirus* and a specific probe for Heartland virus (HRTV) formerly a *Phlebovirus,* now a Banyangvirus were performed. Also, a conventional nested PCR, with the same specific primers was used to detect other *Phleboviruses*, with positive reactions indicated by an amplified cDNA fragment of approximately 244 bp determined by gel electrophoresis. These bands were gel-purified and sequenced by the Sanger method.

**Results:**

Using real-time RT-PCR, no positive results for HRTV were found. However, using conventional nested PCR 2.2% (5/229 pools) yielded a product of 244 bp. One positive sample was detected in a pool of *Dermacentor nitens* ticks collected from a horse, and the four remaining positive pools were from *Rhipicephalus microplus* collected from cattle. The five positive nucleotide sequences had identities of 93 to 96% compared to a section of the L-segment of *Lihan Tick virus*, a *Phlebovirus* originally detected in *R. microplus* ticks in China. The strongest identity (96–99%) was with *Lihan Tick virus* detected in *R. microplus* ticks from Brazil.

**Conclusions:**

This is the first report of viral detection in ticks in Colombia. We detected a Colombian strain of *Lihan Tick virus*. We recommend expanding the sampling area and carrying out more eco-epidemiological studies related to epidemiological surveillance of viruses on ticks in Colombia.

## Synopsis

*Phleboviruses* are RNA viruses transmitted by ticks, phlebotomines and mosquitoes. This study presents the first detection of *Phlebovirus* (*Lihan Tick Virus*) in ticks from Colombia. The sequences detected are related to *Lihan Tick Virus* previously reported in Brazil and China.

## Background

Members of the family Phenuiviridae include human and animal pathogenic viruses transmitted by arthropod vectors, including phlebotomine sandflies, mosquitos and ticks [1, 2]. They can cause a variety of clinical syndromes ranging from a brief, self-limiting febrile illness, to retinitis, encephalitis, meningoencephalitis and fatal haemorrhagic fever [3]. The genus *Phlebovirus* belongs to the family *Phenuiviridae*, order *Bunyavirales,* [4]. The genus includes 10 species with around 58 viruses and several that have not yet been categorized [5]. *Phlebovirus* genomes are single-stranded RNA with negative polarity, organized into three segments. Segment L is approximately 6.4 kb and encodes an RNA-dependent RNA polymerase, segment M is approximately 3.4 kb and encodes viral glycoproteins, and the S segment is approximately 1.7 kb and encodes the nucleocapsid protein. Additional non-stuctural proteins (NSS) are encoded on the S and M segments [3, 6, 7].

In 2018, TBPVs were divided into four phylogenetically related groups: i. the Uukuniemi group, which includes at least 17 species of tick-borne phleboviruses; ii. the severe fever with thrombocytopenia syndrome virus / Heartland virus group; iii. The Bhanja group; and iv. the Kaisodi group. Most of these viruses cause disease in humans [4].

However, in 2019, the group of severe fever with thrombocytopenia syndrome virus / Heartland virus, was taxonomically reclassified in the genus Banyangvirus [5].

In recent years, new TBPVs capable of inducing serious diseases in humans have emerged.

In 2007, the first cases of severe fever with thrombocytopenia syndrome virus (SFTSV), was found in China, later were reported in Japan, Korea and Vietman in patients with severe fever, thrombocytopenia and leukocytopenia accompanied by gastrointestinal symptoms, chills, joint pain and myalgia [8–12]. In the western hemisphere, Heartland virus (HRTV) was first isolated in 2009 from two patients in the state of Missouri, USA, who showed symptoms similar to those of SFTSV. HRTV is phylogenetically related to SFTSV with a similarity of 60 to 70% at the nucleotide level [13]. *Amblyomma americanum* ticks appear to be the main vector for HRTV transmission. To date, more than 30 cases of HRTV infection, including two deaths, have been reported in the United States [14–16]. The status of HRTV in subtropical and tropical regions of the Americas remains unknown.

In Colombia, ticks of the genus *Ixodes, Dermacentor, Amblyomma, Haemaphysalis* and *Rhipicephalus* could act as vectors for a variety of viruses including TBPVs [17]. However, there have been no studies looking for viruses in ticks to date. The aim of this study was to detect and identify HRTV and other TBPVs in ticks collected from domestic animals in different municipalities of the department of Córdoba, Colombia.

## Methods

### Type of study, geographic area and sampling sites

**Fig. 1:**
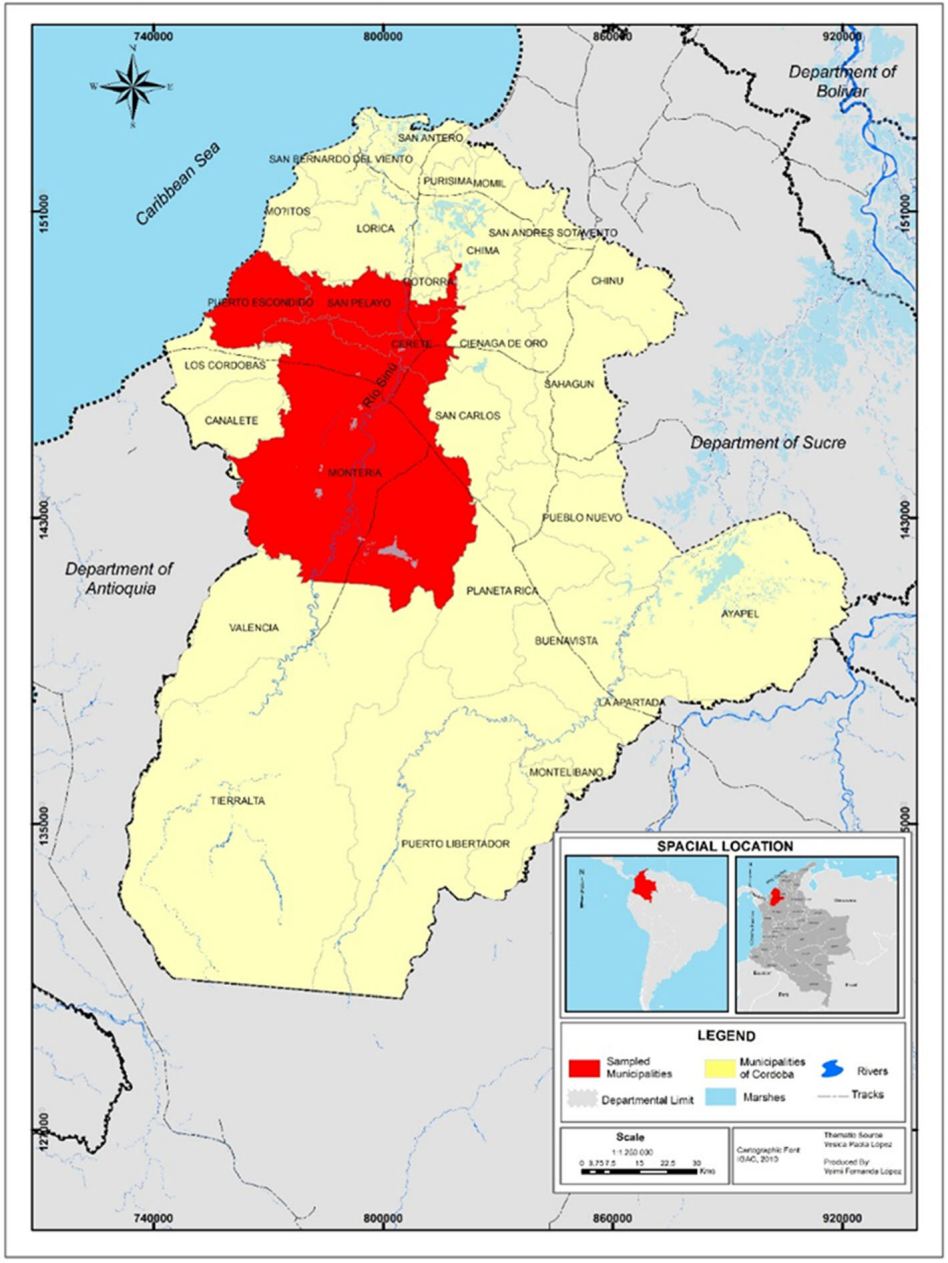
Location of tick collection in four municipalities (Puerto Escondido, San Pelayo, Cerete, Monteria) of the Department of Córdoba

A descriptive and prospective cross-sectional study was conducted between June and December 2016. Ticks attached to dogs, horses, and cattle were collected in the municipalities of Montería, Cereté, San Pelayo and Puerto Escondido in the department of Córdoba, Colombia (Fig. 1). Córdoba is located in the northwestern part of Colombia from 7° 22′ to 9° 26′ north latitude and from 74° 47′ to 76° 30′ west longitude. Córdoba has a warm tropical climate, an average temperature of 28 °C, with altitude ranging from 0 to 40 m above sea level. Its principal economic activity is livestock, agriculture and in grazing, which is over exploited, affecting ecosystems and increasing the burden of ticks potentially responsible for the transmission of many zoonotic diseases [18].

### Ticks collections and taxonomic identification

Hard ticks from the family *Ixodidae* were collected at all developmental stages by removing them from their vertebrate hosts. For storage and transport, ticks were placed in small containers with porous caps for oxygen exchange. Ticks identification was performed using the taxonomic dichotomous keys [19]. Once taxonomically identified, they were frozen at − 90 °C. Ticks were characterized by species, sex and stage and pooled by species, municipality, date and host. The prevalence of *Phlebovirus* in ticks was expressed as a minimum infection rate per 1000 ticks (MIR) based on the assumption that a PCR-positive pool contains at least one positive tick [20].

***Phlebovirus*detection** RNA extraction was carried out with PureLink® RNA Mini Kit combined with TRIzol® (Invitrogen). RNAs were reverse transcribed with Moloney Murine Leukemia Virus Reverse Transcriptase (M-MLV RT), random primers and amplified with nested PCR primers designed to amplify conserved *Phlebovirus* L segment sequence.

For the first round, a conventional PCR was carried out with the genus-specific primers: NPhlebo1f 2054-ATGGARGGITTTGTIWSICIICC-2074 and NPhlebo1r 2295-AARTTRCTIGWIGCYTTIARIGTIGC-2273. For the second round, real-time PCR was used with genus-specific nested primers: NPhlebo2f 2027-WTICCIAAICCIYMSAARATG-2049, NPhlebo2r 2578-TCYTCYTTRTTYTTRARRTARCC-2552 [21] and a FAM-labeled Taqman^Tm^ probe 5′-CGAGTGGAATGGACAGGGAC-BHQ1–3′ for specific detection of HRTV. HRTV RNA was used as positive control. In addition, for the second round a conventional PCR, with the same specific primers was used to detect other *Phleboviruses*.

### Sequencing and phylogenetic analysis

Positive samples were subject to direct sequence of both strands in independent reactions with each of the internal primers and analyzed by the Sanger method (Quintara Biosciences, Inc., Berkeley, CA, USA). Nucleotide sequences were subjected to a BLAST analysis to identify homologous sequences in GenBank. Twenty-six sequences of the L segment of different Phleboviruses were downloaded from GenBank and aligned using MUSCLE in MEGAX. The phylogenetic analysis was performed using the maximum likelihood method with Kimura 2 parameters model with MEGAX [22]..

## Results

A total of 2365 ticks were collected, including 1358 (57.5%) *R. microplus* collected from bovines, 978 (41.3%) *D. nitens* collected from equines, and 29 (1.22%) *Rhipicephalus sanguineus* sensu lato collected from dogs. Ticks were organized in 229 pools.

### Detection of viral RNA in ticks and sequencing analysis

We found that 2.2% of tick pool extracts (5/229) yielded PCR amplicons with the length expected from *Phlebovirus* species (244 bp). One amplicon was detected from 63 pools of *D. nitens* collected from equines (MIR 1.02; 1/978 ticks tested) and the other four amplicons were found among 144 pools of *R. microplus* collected from bovines (MIR of 2.95; 4/1358 ticks tested). None of 29 *R. sanguineus* ticks collected from dogs tested positive. No positive results for HRTV were found.

Alignments of the 244-bp amplicons showed that three of the sequences were identical and the other two sequences had small changes in the nucleotide sequences of 1 and 7 bases.

Sequencing and BLAST analysis showed that the five sequences detected have an identity between 93 to 96% with sequence from the Phlebovirus *Lihan tick virus*, L-segment, originally identified in *R. microplus* ticks in China [23] and identity between 96 to 99% with *Lihan tick virus* detected in *R. microplus* ticks in Brazil in 2018 [24]. Sequences were deposited in GenBank under the following accession numbers: MK040531, MN026336, MN026337, MN026338 and MN026339.

### Phylogenetic analysis

**Fig. 2:**
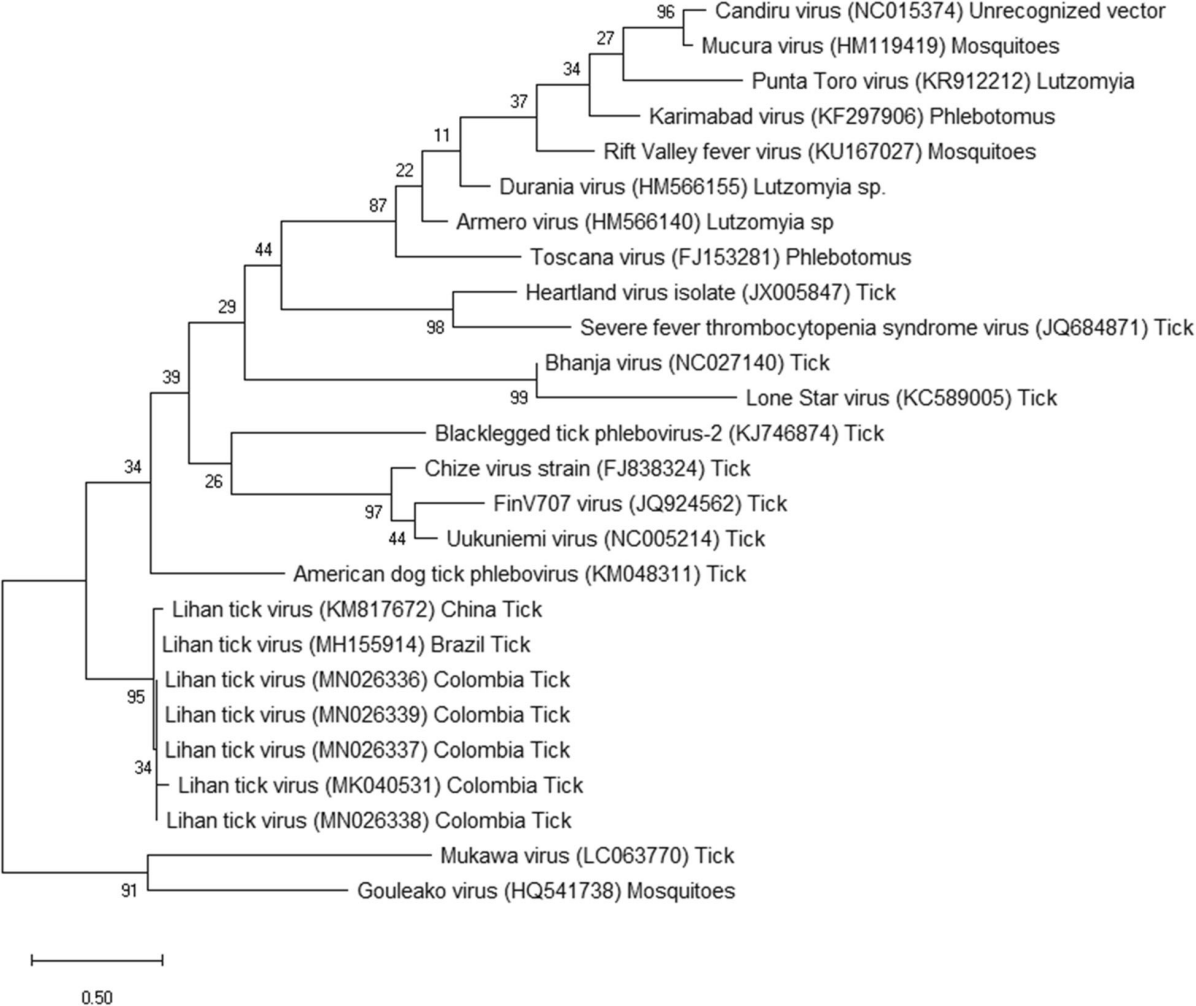
Phylogenetic analysis of Colombian *Lihan Tick virus*. Evolutionary history was inferred by using the Maximum Likelihood method and Kimura 2-parameter model. The tree is drawn to scale, with branch lengths measured in the number of substitutions per site. This analysis involved 26 nucleotide sequences. Bootstrap values were deduced from 1000 replicates. There was a total of 149 positions in the final dataset

A phylogenetic tree was constructed with the five sequences reported in this study and 19 related sequences available in the GenBank, as well as outlier sequences from tick and mosquito-borne viruses, *Mukawa* virus and *Gouléako* virus respectively (Fig. 2). The tick virus sequences detected in this study formed a clade with sequences of *Lihan tick virus* from Brazil and China. These viruses are most similar to other TBPVs and are distant from *Phleboviruses* transmitted by sandflies and mosquitoes.

## Discussion

Ticks are transmitters of many viruses of importance in public health. However, viral diversity in ticks from South America is largely unknown [24]. In recent years the emergence of new TBPV such as SFTSV and HRTV in Asia and North America, respectively, present a significant public health threat [4]. In the present study, TBPV sequences detected in Colombian ticks collected form domestic animals were similar to *Lihan tick virus* detected in China in *R. microplus* ticks [23]. Our results also agree with a work carried out in Southern of Brazil by Souza et al.*,* in 2018, who analyzed six groups of *R. microplus* ticks (~ 50 per group). They used a metagenomic approach with high-throughput sequencing (HTS) and detected *Lihan tick virus* in 5 of 6 groups of ticks [24]. Sameroff et al.*,* 2019, conducted a study in Trinidad and Tobago in 2017 and 2018, in which they analyzed 638 ticks, including *R. microplus* (*n* = 320), *R. sanguineus* (*n* = 300) and *A. ovale* (*n* = 18) with HTS. *Lihan tick virus* was detected in 14 of 16 pools of *R. microplus* (MIR of 43.75; 14/320 ticks tested) results that differ from the MIR of 2.95 (4/1358) reported in our study. However, the methodology used for the detection of the *Lihan tick virus* in these two studies was different, and Sameroff et al. used 20 ticks per pool, whereas we tested pool sizes of 10–15. Furthermore, the stage and sex distribution of ticks and season of collection in the two studies may have differed. These differences as well as geographic variation could explain the variability in the results [25].

A limitation of the present study is the short fragment (less than 200 bp) of the L segment used for phylogenetic analysis. However, this segment is highly conserved and is the most used for phleboviruses phylogenetic studies [21, 26, 27]. Subsequent studies will amplify a larger fragment of the L segment and include the detection of S and M segments, to rule out reassortment of genome segments with other Phenuivirus, in addition to the use of new technologies such as HTS, to better characterize the Colombian viruses. This is the first report of a virus sequence detected in ticks from Colombia and the first Colombian detection of *Lihan tick virus*, a recently described virus with unknown pathogenicity. We suggest that detect of novel viral genomes in Colombia is important and that efforts to establish disease associations with these genomes is essential for One Health.

## Data Availability

The sequences of *Lihan tick virus* have been deposited into GenBank database under the accession numbers: MK040531, MN026336, MN026337, MN026338 and MN026339.

## Abbreviations

TBPV: Tick-borne Phlebovirus
HRTV: Heartland virus
SFTSV: Severe fever with thrombocytopenia syndrome virus
RT-PCR: Reverse transcription polymerase chain reaction
bp: base pairs
MIR: minimum infection rate
